# Glyoxylase-1 combats dicarbonyl stress and right ventricular dysfunction in rodent pulmonary arterial hypertension

**DOI:** 10.3389/fcvm.2022.940932

**Published:** 2022-08-25

**Authors:** Sasha Z. Prisco, Lynn Hartweck, Jennifer L. Keen, Neal Vogel, Felipe Kazmirczak, Megan Eklund, Anna R. Hemnes, Evan L. Brittain, Kurt W. Prins

**Affiliations:** ^1^Cardiovascular Division, Department of Medicine, Lillehei Heart Institute, University of Minnesota, Minneapolis, MN, United States; ^2^Pulmonary and Critical Care, Department of Medicine, University of Minnesota, Minneapolis, MN, United States; ^3^Division of Allergy Pulmonary and Critical Care Medicine, Vanderbilt University Medical Center, Nashville, TN, United States; ^4^Division of Cardiovascular Medicine and Vanderbilt Translational and Clinical Cardiovascular Research Center, Nashville, TN, United States

**Keywords:** right ventricular dysfunction, mitochondria, fatty acid oxidation, gene therapy, metabolism

## Abstract

**Background:**

Heightened glycolytic flux is associated with right ventricular (RV) dysfunction in pulmonary arterial hypertension (PAH). Methylglyoxal, a glycolysis byproduct, is a highly reactive dicarbonyl that has toxic effects *via* non-enzymatic post-translational modifications (protein glycation). Methylglyoxal is degraded by the glyoxylase system, which includes the rate-limiting enzyme glyoxylase-1 (GLO1), to combat dicarbonyl stress. However, the potential consequences of excess protein glycation on RV function are unknown.

**Methods:**

Bioinformatics analysis of previously identified glycated proteins predicted how protein glycation regulated cardiac biology. Methylglyoxal treatment of H9c2 cardiomyocytes evaluated the consequences of excess protein glycation on mitochondrial respiration. The effects of adeno-associated virus serotype 9-mediated (AAV9) GLO1 expression on RV function in monocrotaline rats were quantified with echocardiography and hemodynamic studies. Immunoblots and immunofluorescence were implemented to probe the effects of AAV-Glo1 on total protein glycation and fatty acid oxidation (FAO) and fatty acid binding protein levels.

**Results:**

*In silico* analyses highlighted multiple mitochondrial metabolic pathways may be affected by protein glycation. Exogenous methylglyoxal minimally altered mitochondrial respiration when cells metabolized glucose, however methylglyoxal depressed FAO. AAV9-Glo1 increased RV cardiomyocyte GLO1 expression, reduced total protein glycation, partially restored mitochondrial density, and decreased lipid accumulation. In addition, AAV9-Glo1 increased RV levels of FABP4, a fatty acid binding protein, and hydroxyacyl-CoA dehydrogenase trifunctional multienzyme complex subunits alpha and beta (HADHA and HADHB), the two subunits of the mitochondrial trifunctional protein for FAO. Finally, AAV9-Glo1 blunted RV fibrosis and improved RV systolic and diastolic function.

**Conclusion:**

Excess protein glycation promotes RV dysfunction in preclinical PAH, potentially through suppression of FAO.

## Introduction

RV dysfunction (RVD) is the strongest predictor of mortality in pulmonary arterial hypertension (PAH) (1–5). Unfortunately, there are no currently approved pharmaceuticals that directly augment RV function (6). Thus, a deeper mechanistic understanding of RVD is needed to define potential treatment targets for this lethal consequence of PAH. The most rigorously characterized molecular phenotype of RVD is metabolic remodeling (7). In RV pressure overload, cardiomyocytes exhibit mitochondrial metabolic dysfunction characterized by induction of anaerobic glycolysis, glutaminolysis, and impaired fatty acid oxidation (FAO) (7, 8). In addition, multiple studies demonstrate marked increases in RV glucose uptake in both preclinical PAH models (9) and PAH patients (10–12). Interestingly, RV glucose uptake is strongly and inversely correlated with RV function (10–12), which suggests excess RV glycolysis may have adverse effects on RV performance. However, the secondary consequences of heightened glycolytic flux in RVD are unexplored.

Methylglyoxal is a highly reactive dicarbonyl formed by fragmentation of two glycolytic intermediates: glyceraldehyde-3-phosphate and dihydroxyacetone phosphate (13). As much as 1% of glucose metabolized through glycolysis is converted to methylglyoxal (13). An endogenous methylglyoxal metabolism pathway uses glutathione and two key enzymes, glyoxalase (GLO) 1 and 2 to detoxify methylglyoxal. Initially, methylglyoxal reacts with reduced glutathione to form hemithioacetal. Glyoxylase-1 (GLO1) catalyzes the conversion of hemithioacetal to S-lactoylglutathione, which GLO2 finally converts to D-lactate (14). Excess intracellular methylglyoxal can be toxic as it can modulate protein function by reacting with arginines, lysines, and cysteines (15), a process known as protein glycation. However, protein glycation is partially reversible as DJ-1/PARK7 has deglycase activity (16).

Previous work shows methylglyoxal dysregulation and protein glycation impairs cardiac function *via* depressed mitochondrial function. First, glycation of the ryanodine receptor causes calcium leak which increases mitochondrial calcium levels (17). This in-turn disrupts mitochondrial respiratory activity and induces cardiomyocyte dysfunction (17). Second, knockout of DJ-1 renders mice more susceptible to two cardiac stressors: pressure overload and myocardial infarction (18). Interestingly, DJ-1 knockout mice have lower adenosine triphosphate content and reduced mitochondrial biogenesis (18). Third, viral-mediated overexpression of DJ-1 decreases methylglyoxal levels and protein glycation, and that protects mice from ischemia-reperfusion mediated cardiac failure (19). Finally, with specific regards to the RV, we recently demonstrated small-molecule mediated inhibition of with no lysine kinase-1 suppresses excess protein glycation, improves the RV metabolic signature as demonstrated by normalization of numerous fatty acid metabolites, and ultimately enhances RV function (20). Thus, there are multiple lines of evidence suggesting methylglyoxal-mediated dicarbonyl stress dampens mitochondrial metabolic activity and subsequently depresses cardiac contractility.

Here, we investigated the hypothesis that the excess glycolytic flux that is observed in RV pressure overload (8, 21, 22) leads to heightened protein glycation, likely due to elevated methyglyoxal production, and mitochondrial metabolic impairments that subsequently manifest as worse RV function. We first characterized the effects of methylglyoxal on both glucose and fatty acid metabolism in H9c2 cardiomyoblasts. Then, we examined how adeno-associated virus serotype 9 (AAV9) mediated overexpression of GLO1 impacted protein glycation, the levels of fatty acid binding and oxidizing proteins, RV mitochondrial density, and lipid accumulation in MCT rats. Finally, we determined how AAV9-Glo1 modulated RV anatomy and physiology using histological, echocardiographic, and hemodynamic analyses.

## Materials and methods

### Rat model of pulmonary arterial hypertension

PAH was induced in 200–250 g male Sprague-Dawley rats (Charles River Laboratories, Wilmington, MA) by subcutaneous injection of 60 mg/kg of monocrotaline (Sigma-Aldrich, St. Louis, MO). Control rats received an injection of phosphate buffered saline (PBS). End-point analysis was performed on day 24 after MCT injection. All animal studies were approved by the University of Minnesota Institutional Animal Care and Use Committee.

### Adeno-associated virus serotype 9 production and transduction

AAV9 encoding a cardiac-specific *TNT4* (23) promoter driving FLAG-tagged Glo1 (Supplementary Figure 1) was generated by Vigene Biosciences (Rockville, MD). AAV-green fluorescent protein (GFP) was generated by Professor Xander Wehrens (Supplementary Figure 1) and produced and purchased from the Baylor University Virology Core. One week after MCT injection, rats were randomized to a single intraperitoneal injection of either 1 × 10^11^ vector genomes of AAV-GFP or AAV-Glo1.

### Western blot analysis

Immunoblots were performed on RV specimens as previously described (24) using the Odyssey Infrared Imaging system (LI-COR, Lincoln, NE). Antibodies and dilutions used in this study are described in Supplementary Table 1. Post transfer SDS-PAGE gels were stained with Coomassie brilliant blue and imaged at the 700-nm wavelength on the Odyssey Imaging system and those values were used to normalize values all western blots with LI-COR derived values are show in Supplementary Figure 5.

### Confocal microscopy

RV specimens fixed with 10% formalin were frozen in melting isopentane. 10 μm cryosections were treated with antigen retrieval buffer (10 mM Tris, 1 mM EDTA, 0.05% Tween 20, pH 9.0) and incubated at 95^°^C for 5 min. Samples were then permeabilized with 1% Triton X-100 in PBS for 5 min. Sections were washed/blocked with 5% goat serum in PBS and then incubated with primary antibody for 48 h at 4^°^C. The tissues were then washed/blocked with 5% goat serum and incubated with secondary antibody at 37^°^C for 30 min. For RV cardiomyocyte imaging, formalin fixed RV specimens were manually dissected to isolate fiber bundles. Antigen retrieval was performed by incubating samples in antigen retrieval buffer at 95^°^C for 45 min. Fibers were then permeabilized with 1% Triton X-100 in PBS for 5 min. Cells were then treated with primary antibodies as described above. All samples were embedded in Vectashield containing DAPI (Vector Laboratories, Burlingame, CA). RV cardiomyocytes stained for GLO1 were treated with autofluorescence quenching kit (Vector Laboratories) to reduce mitochondrial autofluorescence. Z-stacks of RV sections and cardiomyocyte bundles were obtained with an Olympus FV1000 BX2 upright confocal microscope (Tokyo, Japan) at the University of Minnesota Imaging Center. All images used to compare fluorescence intensity between groups were collected under identical settings.

### Glyoxylase-1 cardiomyocyte immunoreactivity quantification

RV cardiomyocytes were incubated with GLO1 antibody and processed in FIJI using the threshold function to define the proportion of cells occupied by GLO1 immunoreactivity.

### Mitochondrial density quantification

RV cardiomyocytes were stained with a TOM20 antibody to delineate mitochondria. Images were processed with FIJI and the threshold function was used to quantify the proportion of cardiomyocytes occupied by mitochondria. Area occupied by nuclei was specifically avoided when assessing mitochondrial density.

### FLAG immunoprecipitation

Frozen RV or lung specimens were pulverized in liquid nitrogen and then solubilized in lysis buffer (Pierce, Waltham, MA). BCA assay was performed to determine protein quantification and 500 μg of protein was loaded onto 50 μL of FLAG magnetic beads (Pierce). Bead and extract mixtures were incubated overnight at 4^°^C. Beads were washed with lysis buffer twice and then eluted in low pH buffer. Samples were then mixed with neutralization buffer and saved for later analysis.

### Rodent echocardiography

Echocardiography was performed using a Vevo2100 ultrasound system with a 37.5-MHz transducer (VisualSonics, Toronto, Canada) as we have previously described (24). Representative echocardiography images are presented in Supplementary Figure 2.

### Closed-chest pressure volume loop analysis

A high-fidelity catheter (Scisense 1.9F pressure-volume, Transonic Systems, Ithaca, NY) was placed into the RV *via* the right internal jugular vein in anesthetized rats as we have previously described (20, 25, 26). From PV loop recordings with and without pressure on the inferior vena cava, we determined pulmonary arterial elastance (Ea), RV contractility as represented by end-systolic elastance (Ees), and RV-PA coupling as assessed by Ees/Ea. Representative pressure-volume loops are provided in Supplementary Figure 3.

### H9c2 cell culture

The H9c2 (ATCC CRL-1446) *Rattus norvegicus* cell line were maintained in Dulbecco’s modified Eagle’s medium (DMEM) with 4,500 g/L glucose (ThermoFisher, Waltham, MA), supplemented with 10% heat-treated fetal bovine serum (FBS, GeminiBio, West Sacramento, CA), 100 U/ml penicillin and 100 μg/ml streptomycin (Invitrogen) at 37^°^C with additional 5% CO_2_. Cells were passaged at ∼ 70% confluency with Accutase (BioLegend, San Diego, CA). After 3 days, cells were switched to DMEM with 1% FBS to induce differentiation.

### Seahorse mitochondrial assays

For mitochondrial respiration assays with glucose, the cells were first differentiated by plating 20,000 cells per well of an Agilent XFp Seahorse dish, pre-coated with 0.1% gelatin from porcine skin type A (Sigma-Aldrich). After 3 days, the medium was changed to DMEM 1% FBS. On day five, cells were given fresh Agilent XF DMEM assay medium media either with or without 625 μM methylglyoxal (Sigma-Aldrich). Oxygen consumption rate (OCR) was measured with an Agilent Seahorse XFp Extracellular Flux analyzer. After 3 min of mixing, three 3 min measurements were taken initially to determine the baseline and then after sequential injection of oligomycin A (final concentration after injection 1.5 μM), carbonyl cyanide 4-(trifluoromethoxy) phenylhydrazone (FCCP, 12 μM), and rotenone/actinomycin A (1.0/1.0 μM) (all from Sigma-Aldrich). Data were analyzed using Wave software (Agilent).

For the FAO assay, the cells were first differentiated by plating 20,000 cells per well of an Agilent XFp Seahorse dish, pre-coated with 0.1% gelatin from porcine skin type A (Sigma-Aldrich). After 3 days, the medium was changed to DMEM 1% FBS. On day six, the medium was changed to substrate-limited medium (SLM, Agilent XF DMEM assay medium supplemented with 0.5 mM glucose (Agilent), 1.0 mM glutamine (Agilent), 1% FBS, 25 mM HEPES (Gibco, Waltham, MA) and 0.5 mM carnitine (Sigma-Aldrich). Cells were exposed to SLM with or without 625 μM methylglyoxal (Sigma-Aldrich) for 1 h, then 30 min of assay medium (Agilent XF DMEM assay medium supplemented with 0.5 mM carnitine). Immediately prior to performing the mitochondrial stress assay, BSA-palmitate (Cayman Chemical Company, Ann Arbor, MI) was added to the assay medium to a final concentration of 169 μM. OCR was measured with an Agilent Seahorse XFp Extracellular Flux analyzer. After 3 min of mixing, three 3 min measurements were taken initially to determine the baseline and then after sequential injection of oligomycin A (final concentration after injection 1.5 μM), carbonyl cyanide 4-(trifluoromethoxy) phenylhydrazone (FCCP, 12 μM), and rotenone/actinomycin A (1.0/1.0 μM) (all from Sigma-Aldrich). Data were analyzed with Wave software (Agilent).

### Cardiac histological examination

RV tissues were fixed in 10% formalin, embedded in paraffin, sectioned at 10-μm, and stained with H&E by the University of Minnesota Histology and Research Laboratory in the Clinical and Translational Science Institute. To evaluate cardiomyocyte area, approximately 120 randomly chosen cardiomyocytes from each group (*n* = 3 animals/group) were measured in cross-section at 40 × magnification. RV cryosections at 10-μm were stained with trichrome (Abcam, Cambridge, MA) to quantity fibrosis. Percent fibrosis was assessed by measuring the area of tissue stained blue divided by total tissue area. All measurements were completed in FIJI (Bethesda, MD).

### Oil red O staining and quantification

10-μm RV cryosections were stained with 0.5% Oil Red O (Sigma-Aldrich) in propylene glycol overnight and then counterstained with hematoxylin (Abcam) to identify intramyocardial lipid deposits. Sections were imaged with a Zeiss AxioCam IC and percent Oil Red O stain was determined using FIJI.

### STRING and pathway analysis of previously identified glycated proteins

STRING and pathway analyses of previously published glycated proteins (17) was performed using publically available software.^1^ Pathway analysis (KEGG, Reactome, and Wiki) was conducted on each cluster identified from k-means clustering.

### Statistical analysis

The primary physiological end-point for the preclinical study was Ees/Ea as determined by invasive pressure-volume loop analysis. Secondary end-points included RV ejection fraction (RVEF), RV free wall thickening, TAPSE, tau, and RV end-diastolic pressure (RVEDP). We estimated a sample size of *n* = 15 to reach statistical significance when comparing the three groups. The experiments were terminated before the pre-defined sample size due to the detection of a significant improvement in Ees/Ea.

Statistical analyses were performed with GraphPad Prism v 9.0 (San Diego, CA). Normality of data was evaluated by the Shapiro-Wilk test. If data were normally distributed and if there was equal variance as determined by the Brown-Forsythe test, one-way analysis of variance (ANOVA) with Tukey’s multiple comparisons test was performed. If there was unequal variance, Brown-Forsythe and Welch ANOVA with Dunnett multiple comparisons test were completed when comparing three experimental groups. If the data did not have normal distribution, Kruskal-Wallis test with Dunn’s multiple comparisons test was used when comparing three experimental groups. Two-sided unpaired *t*-test compared the means of two groups if they were normally distributed and the variance was equal as determined by the F-test. Data are presented as mean ± standard error of the mean. Graphs show the mean and all individual values.

## Results

### Bioinformatics analyses linked protein glycation to mitochondrial metabolic function

**FIGURE 1:**
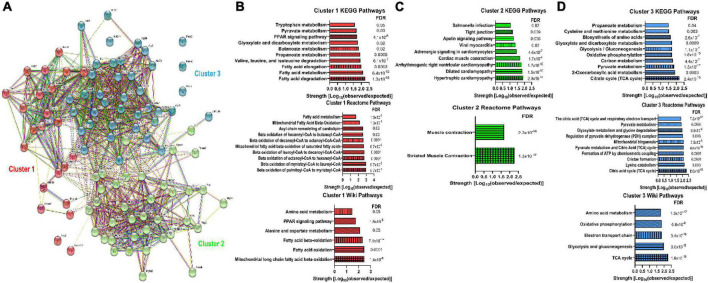
Bioinformatics analysis of previously identified glycated proteins from murine myocardium suggested a link between protein glycation and mitochondrial metabolic activity. **(A)** STRING analysis of the previously identified glycated proteins from murine myocardium (17). K-means clustering delineated three clusters of proteins. **(B)** KEGG pathways, Reactome pathways, and WikiPathways identified in Cluster 1. **(C)** KEGG pathways and Reactome pathways identified in Cluster 2. No WikiPathways were identified from Cluster 2 proteins. **(D)** KEGG pathways, Reactome pathways, and WikiPathways identified in Cluster 3. FDR: False discovery rate.

To evaluate how protein glycation could impact cardiac function, we first performed an *in silico* analysis of previously identified cardiac glycated proteins (17). STRING analysis of glycated proteins was conducted and then we divided the proteins into three groups using k-means clustering (Figure 1A and Supplementary Figure 4). Cluster 1 contained 23 proteins and pathway analyses using three different algorithms identified mitochondrial FAO (Figure 1B). Cluster 2 contained 37 proteins and pathway analyses delineated that cardiomyopathy and muscle contraction (Figure 1C) were linked to this group of proteins. Cluster 3 was comprised of 39 proteins involved in the tricyclic acid cycle, oxidative phosphorylation, and amino acid metabolism (Figure 1D). Thus, our bioinformatics analysis showed two of the three clusters had a strong relationship with mitochondrial metabolic pathways, which suggested protein glycation may impact cardiomyocyte metabolism.

### Dicarbonyl stress compromised fatty acid oxidation in H9c2 cardiomyoblasts

**FIGURE 2:**
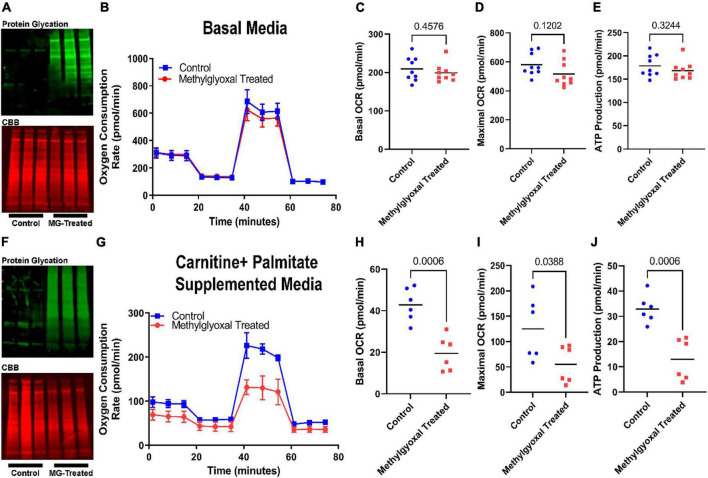
Exogenous methylglyoxal treatment impaired mitochondrial function when H9c2 cardiomyoblasts were exposed to palmitate. **(A)** Western blot of total protein glycation (top) and Coomassie brilliant blue (CBB) SDS-PAGE of control and methylglyoxal treated cells subjected to Mito Stress test. **(B)** Representative Seahorse tracings of cells treated with vehicle or methylglyoxal in the Mito Stress test protocol. Media contained 10 mM glucose, 1 mM pyruvate, and 2 mM glutamine. Quantification of basal oxygen consumption rate **(C)**, maximal oxygen consumption rate **(D)**, and ATP production **(E)**. **(F)** Western blot of total protein glycation (top) and CBB SDS-PAGE (bottom) of control and methylglyoxal treated cells undergoing fatty acid oxidation assay. Media included basal media supplemented with 0.5 mM carnitine and 169 μM palmitate. **(G)** Representative Seahorse tracings of cells treated with vehicle or methylglyoxal in the fatty acid oxidation protocol. Quantification of basal oxygen consumption rate **(H)**, maximal oxygen consumption rate **(I)**, and ATP production **(J)**. *P*-values determined by unpaired *t*-test.

To directly test our *in silico* findings, we first investigated how exogenous methylglyoxal treatment altered H9c2 cardiomyoblast mitochondrial metabolic function using Seahorse analysis. When treated with methylglyoxal, total protein glycation was increased (Figure 2A). However, there were no significant impairments in mitochondrial function when cells predominately metabolized glucose (Figures 2B–E). In contrast, when we interrogated the effect of methylglyoxal when cells were incubated with the fatty acid palmitate, we observed pronounced defects. Methylglyoxal treatment increased total protein glycation and suppressed FAO as there were lower values of basal respiration, maximal respiration, and ATP production (Figures 2F–J). Thus, these results are consistent with a FAO-inhibiting effect of excess methylglyoxal.

### Adeno-associated virus serotype 9-mediated-glyoxylase-1 directed cardiomyocyte glyoxylase-1 overexpression and blunted protein glycation

**FIGURE 3:**
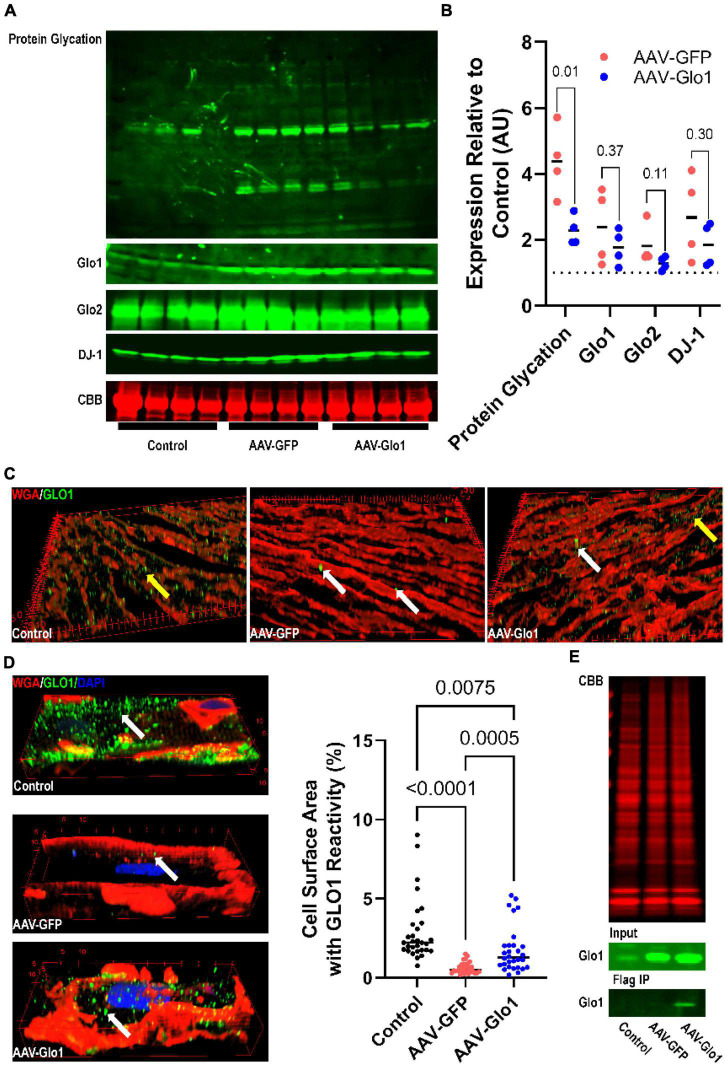
AAV9-mediated GLO1 overexpression reduced protein glycation in the RV of MCT rats. **(A)** Representative Western blot images of protein glycation, GLO1, GLO2, and DJ-1 in RV tissue extracts. Post transfer SDS-PAGE gels were stained with CBB. **(B)** Quantification of Western blot protein expression of protein glycation, GLO1, GLO2, and DJ-1 in the RV. *p*-values determined by unpaired *t*-test because control animals were used as the normalizing standard of 1. **(C)** Three-dimensional reconstructions of RV sections stained with GLO1 antibody. Yellow areas highlight intramyocardial GLO1 staining. AAV-GFP rats had small areas of intense GLO1 immunoreactivity outside of cardiomyocytes (white arrows) and reduced cardiomyocyte GLO1 staining. AAV-Glo1 animals also had punctate staining of high GLO1 reactivity (white arrows), but intramyocardial staining was also increased (yellow arrow). **(D)** Representative three-dimensional projections of confocal micrographs of RV cardiomyocytes showing GLO1 localization. AAV-GFP RV cardiomyocytes had reduced intracellular GLO1 levels, but AAV-Glo1 treatment partially restored cardiomyocyte GLO1 levels. *p*-values determined by Kruskal-Wallis test with Dunn’s multiple comparison test. **(E)** CBB stained SDS-PAGE of input RV extracts for FLAG immunoprecipitation (top). Western blot of GLO1 in input (middle) and FLAG immunoprecipitation elution fraction (bottom) demonstrating FLAG-tagged GLO1 was only detected in AAV-Glo1 animals.

To evaluate the potentially adverse effects of excess protein glycation on RV function, we treated MCT rats with AAV9 encoding either GFP or Glo1. AAV-GFP rats had increased protein glycation (4.4 ± 0.5 fold increase) in RV extracts when compared to control animals, which was significantly mitigated with AAV-Glo1 (2.3 ± 0.2 fold increase compared to controls) (Figures 3A,B). Western blot analysis of RV extracts showed higher levels of GLO1 in both AAV-GFP and AAV-Glo1 rats (Figures 3A,B). To determine the origin of increased GLO1 expression, we evaluated GLO1 immunoreactivity in RV cryosections. GLO1 intracellular signal was detected in control and AAV-Glo1 animals, but the signal was drastically reduced in AAV-GFP animals (Figure 3C). Additionally, there were areas of strong GLO1 immunostaining in extracardiac areas in both AAV-GFP and AAV-Glo1 sections (Figure 3C and Supplementary Videos 1–3), suggesting non-cardiac cells, which express higher levels of GLO1 than cardiomyocytes, contributed to elevated levels of GLO1 in AAV-GFP and AAV-Glo1 total RV extracts. Therefore, we evaluated GLO1 immunoreactivity in mechanically isolated RV cardiomyocytes, and observed a punctate staining pattern at the cell surface in control animals, which was significantly reduced in AAV-GFP animals (Figure 3D and Supplementary Videos 4, 5). However, AAV-Glo1 partially restored RV cardiomyocyte Glo1 immunoreactivity (Figure 3D and Supplementary Video 6). Importantly, FLAG-immunoprecipitation showed FLAG-GLO1 was only detected in AAV-Glo1 rats (Figure 3E), which proved AAV-Glo1 specifically directed expression of GLO1 in RV cardiomyocytes. Finally, GLO2 and DJ-1, proteins responsible for methylglyoxal metabolism and protein deglycation, respectively, were increased in both AAV-GFP and AAV-Glo1 RVs (Figures 3A,B).

### AAV- glyoxylase-1 restored mitochondrial density, increased the abundance of fatty acid binding protein 4/hydroxyacyl-CoA dehydrogenase trifunctional multienzyme complex subunits alpha/beta, and decreased lipid accumulation in the right ventricle of MCT rats

**FIGURE 4:**
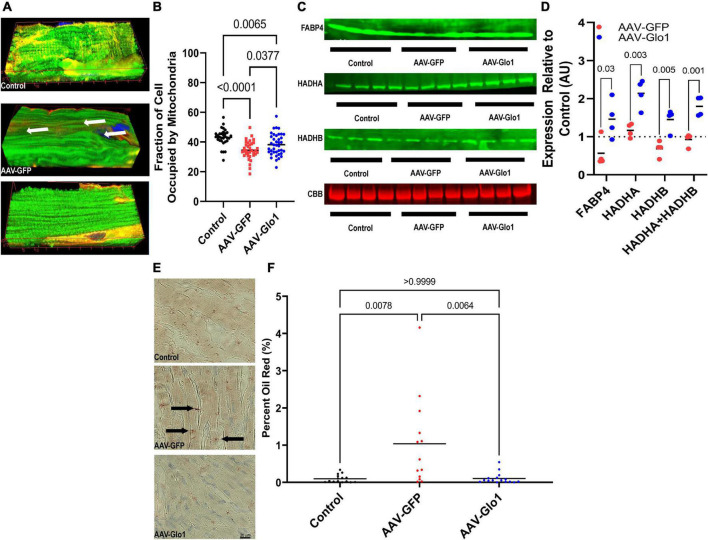
AAV-Glo1 Treatment increased mitochondrial density, the abundance of fatty acid handling and metabolic proteins, and prevented lipid accumulation in the RV of MCT rats. **(A)** Representative three-dimensional confocal micrographs of RV cardiomyocytes stained with the mitochondrial marker TOM20 (green) and wheat germ agglutinin (red) to outline cells. Arrows highlight areas of reduced mitochondrial density. **(B)** Quantification of mitochondrial density in RV cardiomyocytes. *p*-values determined by one-way ANOVA with Tukey’s multiple comparisons test. **(C)** Representative Western blots and quantification **(D)** of protein abundance of fatty acid handling and metabolizing proteins FABP4, HADHA, and HADHB. *p*-values determined by unpaired *t*-test with control values serving as a reference of 1. *n* = 3 control for HADHB but *n* = 4 for controls for FABP4 and HADHA. **(E)** Representative images of RV sections subjected to Oil Red O staining. Arrows highlight intramyocardial fatty acid detection. Scale bar 40 μm. **(F)** Quantification of Oil Red O staining in RV sections. *p*-values determined by Kruskal-Wallis test with Dunn’s multiple comparison test.

To examine how modulation of dicarbonyl stress impacted RV mitochondrial structure and function, we used confocal microscopy to probe RV cardiomyocyte mitochondria density. As compared to control RV cardiomyocytes, there was a significant reduction in mitochondrial density in AAV-GFP specimens. However, AAV-Glo1 treatment increased RV cardiomyocyte mitochondrial density (Control: 43.1 ± 1.0%, AAV-GFP: 34.5 ± 1.0%, AAV-Glo1: 38.3 ± 1.2% of cell area) (Figures 4A,B and Supplementary Videos 7–9). To determined how protein glycation may modulate fatty acid handling and FAO proteins, we probed the levels of fatty acid binding protein 4 (FABP4) and hydroxyacyl-CoA dehydrogenase trifunctional multienzyme complex subunits alpha and beta (HADHA and HADHA) in RV extracts. AAV-Glo1 treatment significantly increased levels of FABP4, HADHA, and HADHB in the RV when compared to AAV-GFP (Figures 4C,D). Because HADHA and HADHB function as a heterotetramer to facilitate FAO (27), we evaluated how their combined expression was modulated by AAV-Glo1 treatment and observed a significant increase as compared to AAV-GFP animals (Figures 4C,D). Then, we performed Oil Red O staining to quantify lipid accumulation to evaluate for signs of impaired RV lipid metabolism. Intramyocardial lipid deposition was higher in AAV-GFP RVs as compared to controls, but AAV-Glo1 rats had significantly less RV lipid accumulation (Control: 0.1 ± 0.03%, AAV-GFP: 1.0 ± 0.3%, AAV-Glo1: 0.1 ± 0.04%) (Figures 4E,F).

### Glyoxylase-1 overexpression reduced right ventricular fibrosis

**FIGURE 5:**
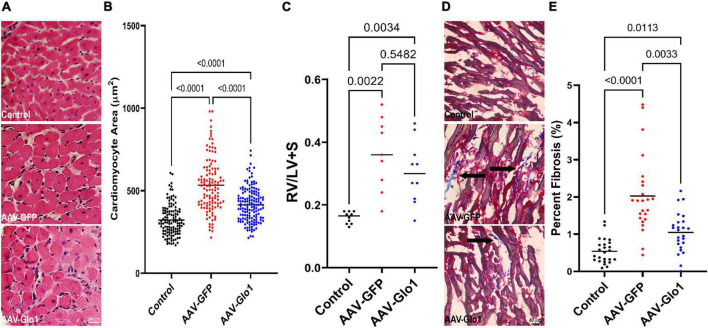
AAV-Glo1 had a modest effect on RV hypertrophy but reduced RV fibrosis. **(A)** Representative images of hematoxylin and eosin-stained RV sections. Scale bar 20 μm. **(B)** Quantification of RV cardiomyocyte cross-sectional area. Glo1 overexpression reduced RV cardiomyocyte area. **(C)** Quantification of RV to LV + septum mass. *p*-values determined by one-way ANOVA with Tukey’s multiple comparisons test **(D)** Representative images of Masson-Trichrome stained RV sections to delineate fibrosis (marked by arrows). Scale bar 40 μm. **(E)** Quantification of RV fibrosis area. AAV-Glo1 treatment reduced RV fibrosis. *p*-values determined by Kruskal-Wallis test with Dunn’s multiple comparison test in **(B,E)**.

Subsequently, we assessed how GLO1 overexpression modulated RV hypertrophy and fibrosis. At the cardiomyocyte level, AAV-Glo1 rats had a slight reduction in cardiomyocyte cross-sectional area as compared to AAV-GFP rats (Control: 322 ± 9 μm, AAV-GFP: 534 ± 15 μm, AAV-Glo1: 415 ± 9 μm), but cardiomyocyte size was not normalized (Figures 5A,B). At the organ level, the ratio of RV to left ventricle plus septum was not significantly reduced with AAV-Glo1 (Figure 5C), which implied Glo1 had a minor effect on RV hypertrophy. On the other hand, RV fibrosis was heightened in AAV-GFP rats, but it was mitigated by AAV-Glo1 treatment (Control: 0.5 ± 0.07%, AAV-GFP: 2.0 ± 0.2%, AAV-Glo1: 1.0 ± 0.1%) (Figures 5D,E). Thus, AAV-Glo1 treatment predominately modulated RV fibrosis and not hypertrophy.

### Adeno-associated virus serotype 9-mediated-glyoxylase-1 improved right ventricular function independent of pulmonary vascular disease severity

**FIGURE 6:**
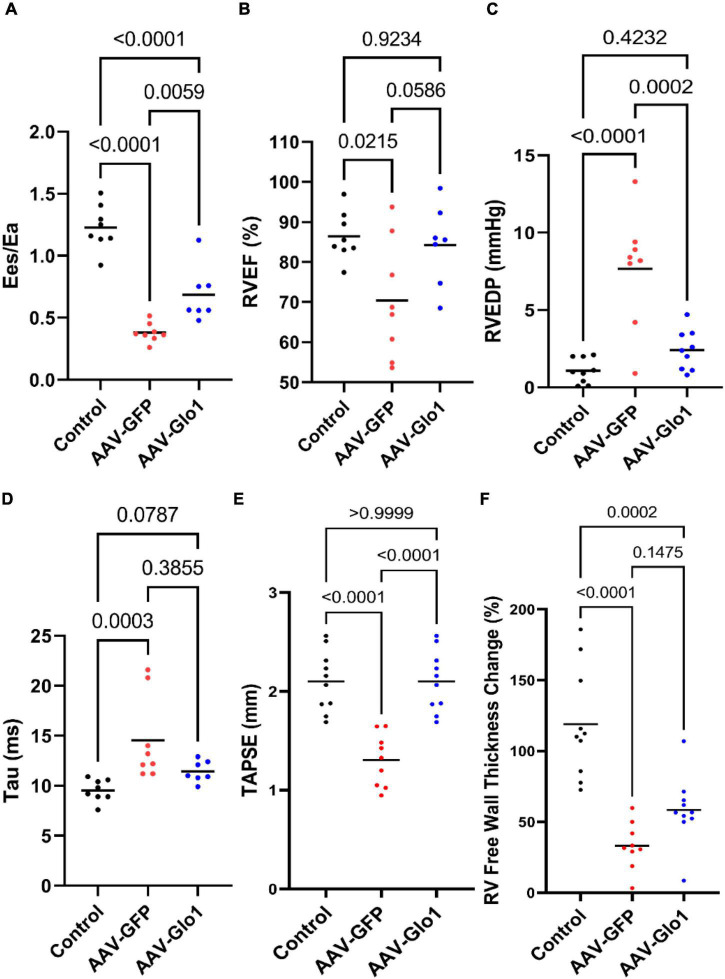
GLO1 overexpression enhanced RV systolic and diastolic function. **(A)** AAV-Glo1 treatment significantly improved Ees/Ea. AAV-Glo1 non-significantly improved RV ejection fraction (RVEF) **(B)** and significantly reduced RVEDP **(C)** compared to AAV-GFP. **(D)** RV tau was non-significantly reduced by AAV-Glo1. **(E)** TAPSE was normalized by AAV-Glo1 treatment and **(F)** RV free wall thickening was augmented, although not statistically different from AAV-GFP animals. *p*-values determined by using one-way ANOVA with Tukey’s multiple comparisons test in **(A–C,E,F)** and Kruskal-Wallis test with Dunn’s multiple comparison test in **(D)**.

Pressure-volume loop analysis demonstrated GLO1 overexpression significantly improved the primary outcome in this study: RV-pulmonary arterial coupling (Ees/Ea) (Control: 1.2 ± 0.1, AAV-GFP: 0.4 ± 0.03, AAV-Glo1: 0.7 ± 0.1) (Figure 6A). Consistent with this finding, AAV-Glo1 rats had higher RVEF (Control: 86 ± 2%, AAV-GFP: 70 ± 5%, AAV-Glo1: 84 ± 4%) (Figure 6B), but the RVEF values between AAV-GFP and AAV-Glo1 were not statistically different (*p* = 0.06). RVEDP was increased in AAV-GFP rats, but GLO1 overexpression normalized RVEDP (Control: 1.1 ± 0.3 mm Hg, AAV-GFP: 7.7 ± 1.3 mm Hg, AAV-GLO1: 2.4 ± 0.4 mmHg) (Figure 6C). Tau, a measure of RV diastolic function, was elevated in AAV-GFP as compared to controls, however GLO1 overexpression manifested as a non-significant reduction as compared to AAV-GFP (Control: 9.5 ± 0.4 ms, AAV-GFP: 14.5 ± 1.5 ms, AAV-Glo1: 11.4 ± 0.4 ms) (Figure 6D).

Echocardiography corroborated our pressure-volume loop analysis as there was a significant reduction in (tricuspid annular plane systolic excursion) TAPSE in the AAV-GFP compared to control, but AAV-Glo1 normalized TAPSE values (Control: 2.1 ± 0.1 mm, AAV-GFP: 1.3 ± 0.1 mm, AAV-Glo1: 2.1 ± 0.1 mm) (Figure 6E). Finally, RV free wall thickening was lower in AAV-GFP compared to controls, and there was a non-significant improvement with GLO1 overexpression (Control: 118.9 ± 12.2%, AAV-GFP: 33.2 ± 5.5%, AAV-Glo1: 58.4 ± 7.6%) (Figure 6F).

**FIGURE 7:**
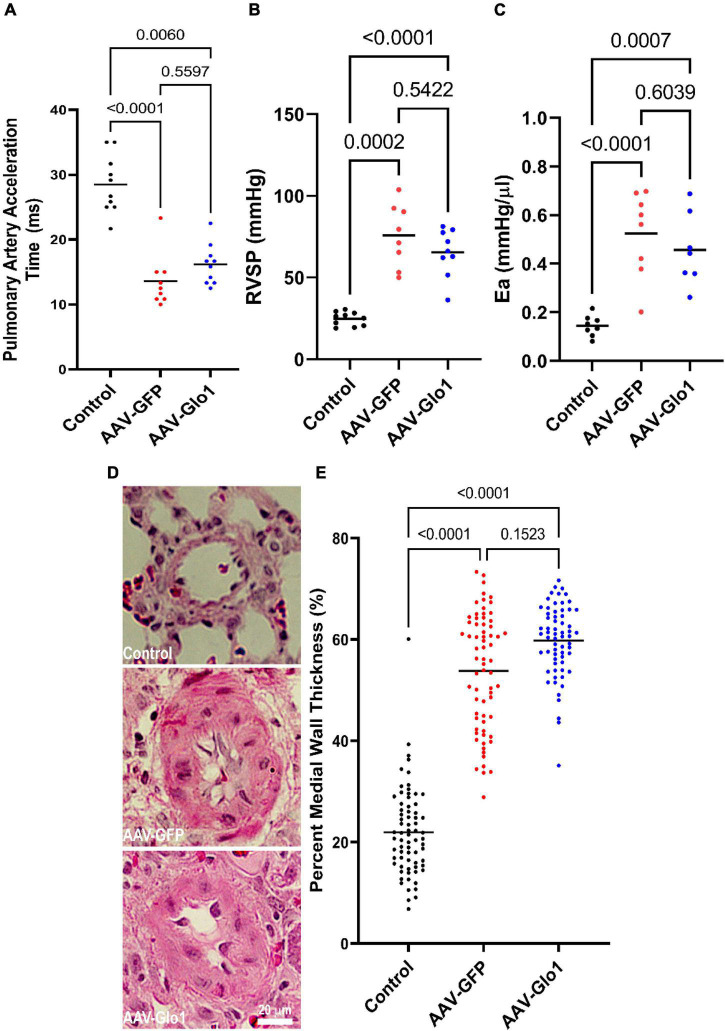
GLO1 overexpression did not alter PAH severity. Pulmonary artery acceleration time **(A)**, RV systolic pressure (RVSP) **(B)**, and effective arterial elastance (Ea) **(C)** were all equivalent in AAV-GFP and AAV-Glo1 rats. **(D)** Representative images of hematoxylin and eosin stained small pulmonary arterioles. Scale bar 20 μm. **(E)** Quantification of small arteriole remodeling in lung sections. *p*-values determined by Kruskal-Wallis test with Dunn’s multiple comparison test in **(A,E)**, Brown-Forsythe and Welch ANOVA with Dunnett multiple comparisons test in **(B)**, and one-way ANOVA with Tukey’s multiple comparisons test in **(C)**.

Importantly but not unexpected, as Glo1 was not overexpression in the pulmonary vasculature (Supplementary Figure 6) there were no differences in multiple parameters of pulmonary vascular disease severity between AAV-GFP and AAV-Glo1 rats. Pulmonary artery acceleration time (Control: 28.5 ± 4.5 ms, AAV-GFP: 13.6 ± 4.0 ms, AAV-Glo1: 16.2 ± 3.1 ms), RV systolic pressure (Control: 24.8 ± 1.3 mmHg, AAV-GFP: 75.8 ± 6.8 mmHg, AAV-Glo1: 65.5 ± 4.8 mmHg), and effective arterial elastance (Control: 0.14 ± 0.02 mmHg/μl, AAV-GFP: 0.52 ± 0.06 mmHg/μl, AAV-Glo1: 0.46 ± 0.05 mmHg/μl) were comparable between AAV-GFP and AAV-Glo1 (Figures 7A–C). Finally, percent medial wall thickness (Control: 21.9 ± 1.0%, AAV-GFP: 53.8 ± 1.4%, AAV-Glo1: 59.8 ± 0.9%) was equivalent in AAV-GFP and AAV-Glo1 animals (Figures 7D,E). In summary, these data suggest AAV-Glo1 enhanced RV function without significantly altering pulmonary vascular disease severity.

## Discussion

In this current work, we show exogenous methylglyoxal specifically depresses mitochondrial function in H9c2 cardiomyoblasts supplemented with fatty acid, consistent with our *in silico* evaluation showing multiple FAO proteins are glycated. In MCT rats treated with AAV-Glo1, total RV protein glycation is reduced by approximately 50%, mitochondrial density is increased, fatty acid handling and FAO proteins levels are elevated, and pathological lipid accumulation is mitigated. In addition, Glo1 overexpression combats RV fibrosis and echocardiography and closed-chest pressure volume loop analysis show AAV-Glo1 treatment enhances RV systolic and diastolic function without significantly altering PAH severity. In summary, these results suggest a new link between dicarbonyl-mediated protein glycation, impaired FAO, and depressed RV function in a rodent model of PAH. To the best of our knowledge, this is the first use of gene therapy to specifically enhance RV function in preclinical model PAH, suggesting a novel therapeutic approach for this uniformly lethal disease. Moreover, these data provide evidence that RV function can be improved even in the setting of significant pulmonary hypertension, which supports the notion that the RV can be a viable therapeutic target in PAH (8).

Our work provides further support for the findings that excess methylglyoxal production and/or impaired dicarbonyl metabolism promotes cardiac dysfunction under multiple cardiac stressors. As discussed above, ablation of the deglycase enzyme DJ-1 exacerbates cardiac dysfunction due to ischemic injury or pressure overload (18) while overexpression of DJ-1 mitigates compromised systolic function in both ischemia and pressure overload (19). Importantly, modulation of DJ-1 either accentuates or combats mitochondrial dysfunction in these settings. Thus, these findings supports the hypothesis that excess protein glycation impairs cardiac metabolism. In addition, Glo1 transgenic mice are partially protected from myocardial infarction-induced cardiac dysfunction due to reduced cardiac inflammation and improved vascular density (28). Finally, protein glycation compromises sarcomere function in both rodent and human models of diabetic cardiomyopathy (29, 30). Thus, the cardiotoxic effects of methylglyoxal accumulation and subsequent protein glycation are diverse and include blunted mitochondrial metabolic function, cardiac inflammation, reduced vascular density, and depressed sarcomeric force generation.

Our finding that methylglyoxal treatment impaired FAO *in vitro* is supported by the observation that proteins responsible for multiple aspects of fatty acid handling and metabolism are proposed to be glycated. Interestingly, many of these proteins are already associated with RV dysfunction in PAH. First, fatty acid binding proteins (FABP) 3 and 4, which are responsible for intracellular shuttling of fatty acids, were identified as protein glycation targets in a previous proteomics study (17). FABP4 is downregulated in the RV of the Sugen-hypoxia PAH model and in PAH patients with RV dysfunction (22), and thus it may be directly relevant to compromised RV function in PAH. We show AAV-Glo1 increases levels of FABP4, which may help explain the reduction in RV lipid accumulation with GLO1 overexpression. Interestingly, the peroxisome proliferator-activated receptor gamma agonist pioglitazone also restores FABP4 levels and augments RV function in the Sugen-hypoxia model of PAH (22), which suggests combatting FABP4 downregulation may have therapeutic relevance for RV failure. In addition, we show GLO1 overexpression increases the levels of both subunits of the mitochondrial trifunctional protein (HADHA and HADHB), which implies glycation of these proteins may alter their stability and subsequently impair FAO. This could be another mechanism underlying lipid accumulation observed in the RV in PAH (26, 31). Finally, carnitine O-acetyltransferase (CRAT), an enzyme essential for import of acylcarnitines into the mitochondria for subsequent degradation, was also identified in the proteomics screen. Perhaps glycation of CRAT contributes to the impaired acylcarnitine metabolism that is observed in both preclinical and human PAH-associated RV dysfunction (20, 25, 31).

Our manuscript adds to a growing literature linking alterations in GLO1 to impaired fatty acid metabolism in multiple organisms and in distinct tissues. In zebrafish Glo1 knockouts, there is evidence of impaired fatty acid metabolism as demonstrated by elevated levels of multiple lipid species (32). Moreover, Glo1 knockout zebrafish exhibit hepatic lipid accumulation when subjected to high fat diet (32). *Drosophila* Glo1 knockouts also show signs of impaired FAO with increased triglyceride accumulation (33). In the liver, downregulation of GLO1 is associated with the development of non-alcoholic fatty liver disease in mice and humans (34). The summation of these data show GLO1 dysregulation modulates fatty acid metabolism, and they may provide additional insights into other etiologies of cardiac disease. In particular, it is possible that modulation of methylglyoxal metabolism could contribute to the consistent observation that diabetic cardiomyopathy is associated with lipotoxicity (35, 36). In summary, we hypothesize increased methylglyoxal production impairs FAO, and this could be another molecular mechanism underlying the Randle cycle, the observation that glucose metabolism and FAO are inversely associated (37, 38).

Finally, our results implicating the toxic effects of methylglyoxal may be even more relevant in RVD than in left ventricular dysfunction. First, isolated RV cardiomyocytes exhibit higher rates of glycolysis than left ventricular cardiomyocytes (39), suggesting there may be more methylglyoxal production in RV cardiomyocytes at baseline. Furthermore, analysis of the Human Heart Atlas (40) shows Glo1 mRNA levels are higher in the RV in group 2 and 3 cardiomyocytes. Importantly, group 2 cardiomyocytes are more prevalent in the RV than LV (41) and thus Glo1 may be even more important for the RV (Supplementary Table 2). In addition, DJ-1 and Glo2 mRNAs are more highly expressed in group 2 RV cardiomyocytes (Supplementary Table 2). These mRNA findings are also consistent with differences in protein abundance observed in multiple species. Evaluation of the online proteomics database atlas.cardiacproteomics.com (42) shows *Rattus norvegicus*, *Mus musculus*, and *Equus caballus* all have higher GLO1 levels in the RV than LV (Supplementary Table 3).

### Limitations

Our study has important limitations that we must acknowledge. First, we only evaluated the therapeutic efficacy of AAV-Glo1 in male rats because female MCT rats do not develop significant RV dysfunction. Not all measures of RV function were significantly improved with AAV-Glo1 treatment, which may be due to sample size. We stopped the study early because we reached our primary end-point, but it is possible that by increasing our sample size we could have achieved statistical significance in more or all RV functional parameters. We were unable to get adequate volumes from all animals during pressure-volume loop analysis experiments despite multiple catheter position changes or because the internal jugular vein tore (*n* = 1) and we had to abort the procedure. The presence of non-cardiac cells in our whole RV extracts likely impacted our Western blot results when evaluating GLO1, GLO2, and DJ-1. Consistent with this hypothesis, GLO1 proteins levels are predicted to be very low in cardiomyocytes in the human protein atlas^2^ and single-cell RNA sequencing analysis shows immune cells have higher levels of GLO1 transcript than cardiomyocytes.^3^ Finally, we observed slightly more GLO1, GLO2, and DJ-1 in our MCT RV extracts as compared to our previous results (20). However, we suspect this may be due to immune cell infiltration as AAV triggers an immune response (43).

## Data availability statement

The data presented in this study are deposited in the GenBank repository, accession number 2606819.

## Ethics statement

The animal study was reviewed and approved by the University of Minnesota IACUC.

## Author contributions

All authors listed have made a substantial, direct, and intellectual contribution to the work, and approved it for publication.

## Footnotes

1
https://string-db.org/cgi/input?sessionId=bTCCsEzBV1cL

2
https://www.proteinatlas.org/ENSG00000124767-GLO1/tissue

3
https://www.proteinatlas.org/ENSG00000124767-GLO1/single+cell+type/heart+muscle
