# Supplementary material for: Glyoxylase-1 combats dicarbonyl stress and right ventricular dysfunction in rodent pulmonary arterial hypertension
Source: Front Cardiovasc Med. 2022 Aug 25;9:940932. doi: 10.3389/fcvm.2022.940932 (PMC9452736; doi:10.3389/fcvm.2022.940932)

Supplemental Table 1. Antibodies used in the present study.

**Antigen**	**Company**	**Species**	**Catalogue Number**	**Experimental Approach**	**Dilution**
DJ-1	Abcam	Rabbit	ab18257	Western blot	1:250
GLO1	Abcam	Rabbit	ab129124	Immunofluorescence	1:50
GLO1	Abcam	Mouse	ab171121	Western blot	1:200
GLO2	Thermo Fisher Scientific	Rabbit	PA5-93097	Western blot	1:250
FABP4	Abcam	Rabbit	ab92501	Western blot	1:250
HADHA	Abcam	Rabbit	ab203114	Western blot	1:250
HADHB	Abcam	Rabbit	ab230667	Western blot	1:250
TOM20	Cell Signaling Technology	Rabbit	42406	Immunofluorescence	1:50
Methylglyoxal	Cell Biolabs	Mouse	STA-011	Western blot	1:100
Alexa fluor 566 anti-rabbit secondary	Invitrogen	Goat	A32732	Immunofluorescence	1:500
Anti-mouse secondary	LI-COR	Goat	926-32210<br>926-68070	Western blot	1:5000
Anti-rabbit secondary	LI-COR	Goat	926-32211<br>926-68071	Western blot	1:5000
Alexa fluor-633 conjugated Wheat Germ Agglutinin	Invitrogen		W21404	Immunofluorescence	1:50

Supplemental Table 2: Analysis of mRNA levels of DJ-1, Glo1, and Glo2 in human LV and RV cardiomyocytes from the Human Heart Atlas. The RV has a higher proportion of group 2 cardiomyocytes (vCM2_RV) than the LV (39.91 vs. 9.12%), suggesting that this population of cardiomyocytes may have a more important role of RV function than LV function.

Gene	vCM1_LV	vCM1_RV	vCM2_LV	vCM2_RV	vCM3_LV	vCM3_RV	vCM4_LV	vCM4_RV	vCM5_LV	vCM5_RV
**DJ-1**	0.248	0.244	0.229	**0.245**	0.273	0.211	0.545	0.434	0.253	0.245
**Glo1**	0.238	0.237	0.231	**0.234**	0.206	**0.257**	0.237	0.183	0.298	0.211
**Glo2**	0.514	**0.570**	0.509	**0.523**	0.419	**0.537**	0.516	0.474	0.641	0.565

Supplemental Table 3: Proteomic-based analysis of protein abundance of DJ-1, GLO-1, and GLO-2 in RV and LV of three different species. Data obtained from the online cardiac proteomics database, which has cardiac chamber protein signatures across multiple mammalian species. There are three biological replicates for protein intensity for each protein per ventricle tested. The ventricles with the highest expression of each protein per animal are bolded.

Organism	Protein	RV	LV
Rattus norvegicus			
	DJ-1	**2.0E10, 2.4E10, 1.6E10**	1.8E10, 1.8E10, 1.6E10
	GLO1	**5.3E9, 4.1E9, 4.1E9**	5.2E9, 4.0E9, 2.8E9
	GLO2	**1.2E9, 1.7E9, 1.7E9**	1.4E9, 1.4E9, 1.4E9
Mus musculus			
	DJ-1	1.3E10, 1.4E10, 1.4E10	**1.6E10, 1.7E10, 1.3E10**
	GLO1	**5.3E9, 6.6E9, 6.8E9**	5.4E9, 5.6E9, 3.9E9
	GLO2	2.7E9, 2.6E9, 2.3E9	**4.1E9, 3.2E9, 3.1E9**
Equus caballus			
	DJ-1	**2.6E10, 2.8E10, 1.4E10**	2.6E10, 2.0E10, 2.3E10
	GLO1	**6.7E9, 7.1E9, 8.7E9**	7.3E9, 6.4E9, 6.5E9
	GLO2	**3.5E9, 4.0E9, 3.5E9**	2.9E9, 3.9E9, 3.2E9

Supplemental Figure 1: AAV-GFP and AAV-Glo1 plasmids used to generate the AAV vectors in this study.

pAAV9-Glo1

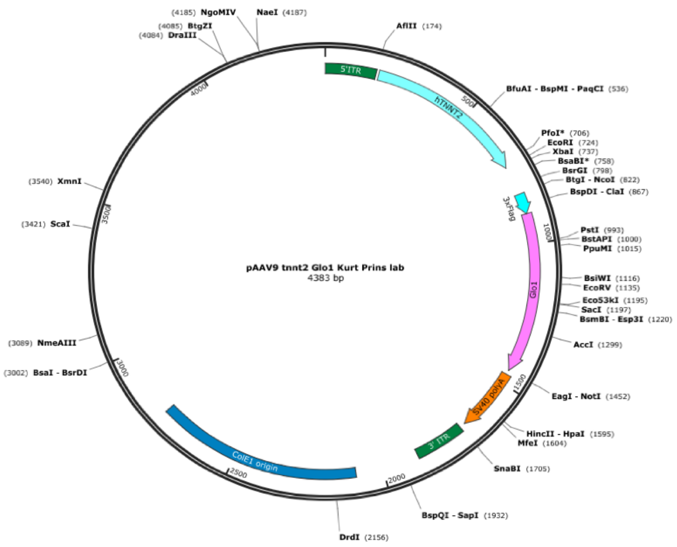

pAAV9-GFP

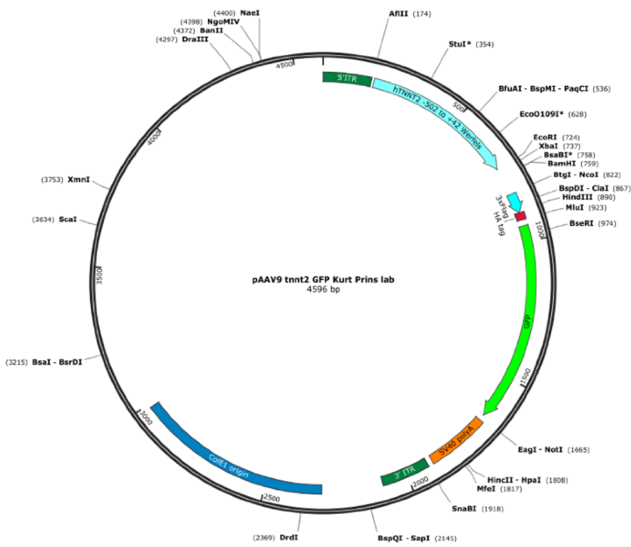

Supplemental Figure 2: Representative echocardiography images from rodent studies.

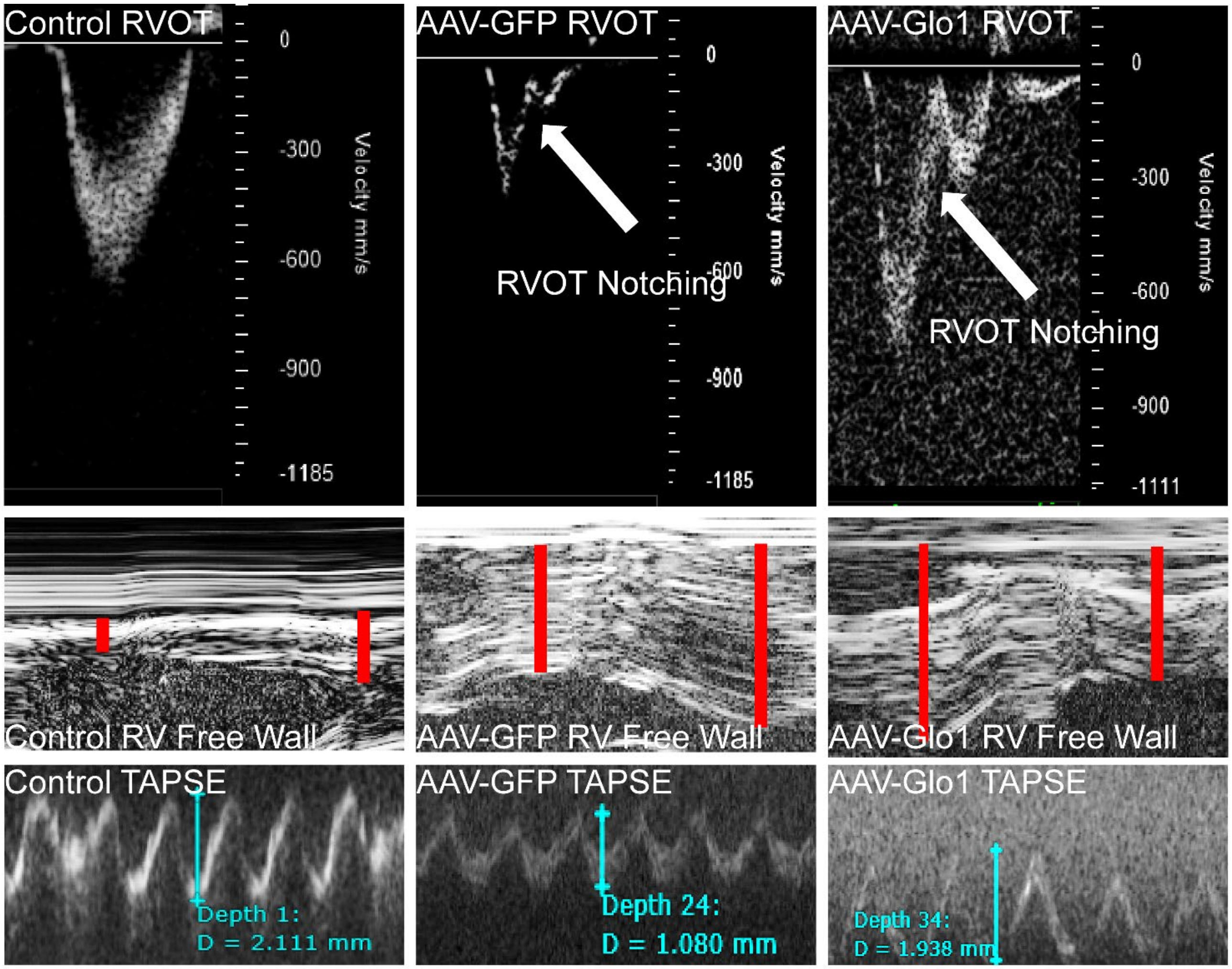

Supplemental Figure 3: Example PV loops and end-systolic elastance (Ees) lines from control, AAV-GFP, and AAV-Glo1 rats.

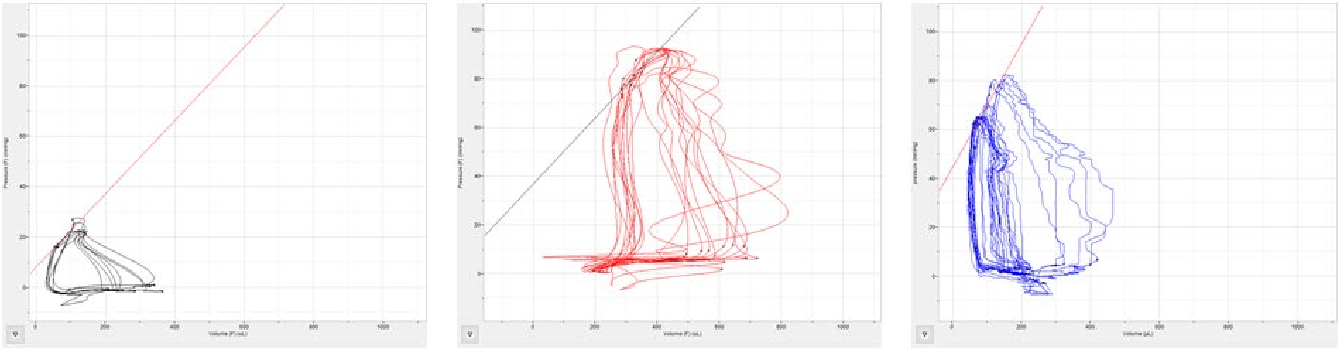

Control AAV-GFP AAV-Glo1

Supplemental Figure 4: Images of Cluster 1 (A), Cluster 2 (B), and Cluster 3 (C) from the STRING analysis of previously identified glycated proteins from Figure 1.

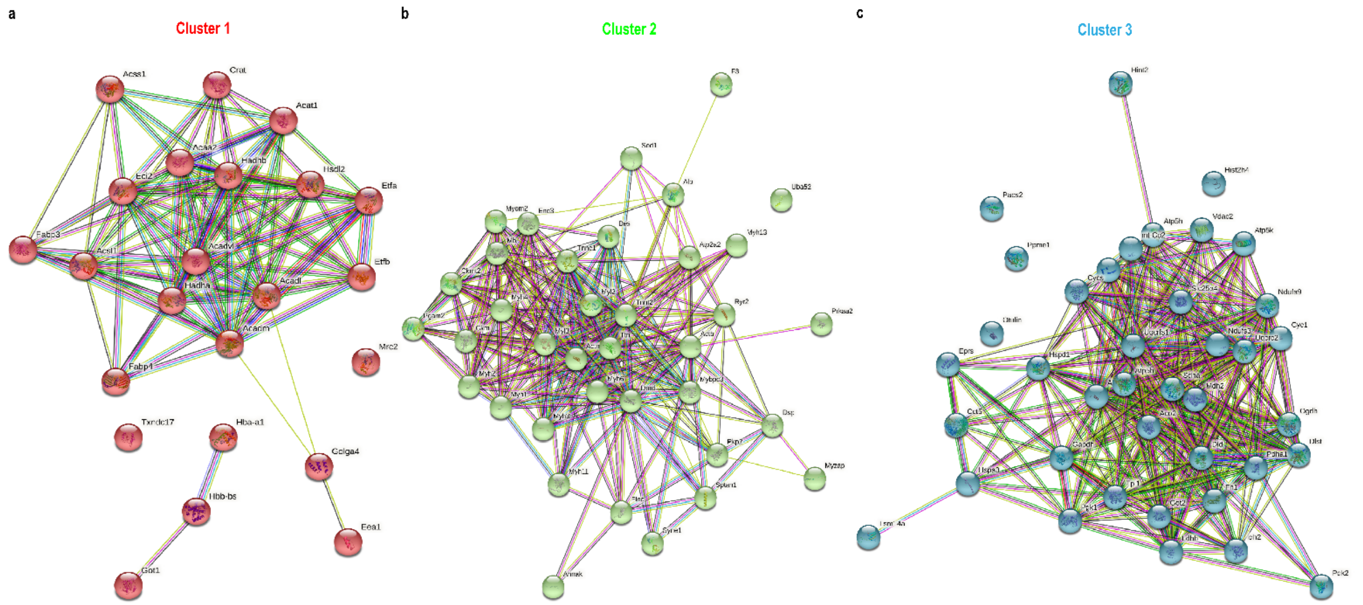

Supplemental Figure 5: Western blots with quantification values derived from the Li-Cor software presented in Figure 3 and 4.

Protein Glycation

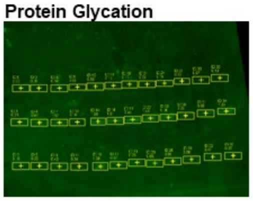

GLO1

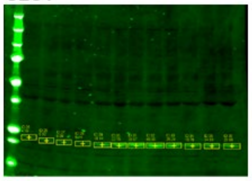

Glo2

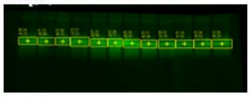

DJ-1

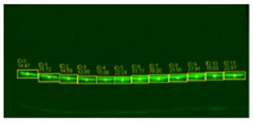

CBB Loading Control

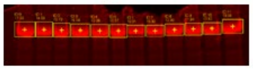

FABP4

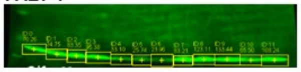

HADHA

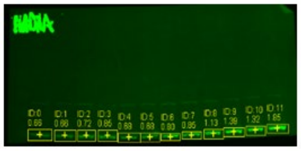

HADHB

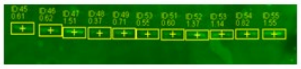

Supplemental Figure 6: Flag-tagged Glo1 was not detected in lung extracts in animals treated with AAV-Glo1 virus. (A) CBB stained SDS-PAGE of start, void, and elution fraction of lung extracts exposed to Flag-antibody from control and AAV-Glo1 treated animals. (B) Western blot of start, void, and elution fraction of lung extracts. Glo1 is detected in the start and void fractions from both animals, but not in the elution fraction. Thus, Flag-tagged Glo1 was not detected in the lung.

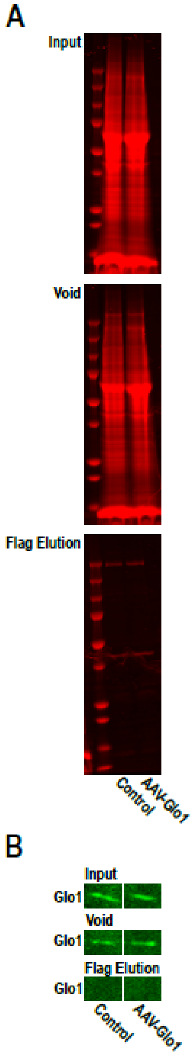

Supplement: Supplementary file 1 [file Data_Sheet_1.pdf]
